# Development of a visual Adhesion/Invasion Inhibition Assay to assess the functionality of *Shigella*-specific antibodies

**DOI:** 10.3389/fimmu.2024.1374293

**Published:** 2024-04-12

**Authors:** Giampiero Batani, Giacomo Vezzani, Sabrina Lashchuk, Abdelmounaaim Allaoui, Dario Cardamone, Maria Michelina Raso, Elena Boero, Emanuele Roscioli, Matteo Ridelfi, Gianmarco Gasperini, Mariagrazia Pizza, Omar Rossi, Francesco Berlanda Scorza, Francesca Micoli, Rino Rappuoli, Claudia Sala

**Affiliations:** ^1^ Monoclonal Antibody Discovery Laboratory, Fondazione Toscana Life Sciences, Siena, Italy; ^2^ GlaxoSmithKline (GSK) Vaccines Institute for Global Health (GVGH), Siena, Italy; ^3^ The Microbiology Laboratory, University Mohammed VI Polytechnic, Ben, Guerir, Morocco; ^4^ Data Science for Health Laboratory, Fondazione Toscana Life Sciences, Siena, Italy; ^5^ Department of Life Sciences, Imperial College, London, United Kingdom; ^6^ Fondazione Biotecnopolo di Siena, Siena, Italy

**Keywords:** *Shigella* pathogenesis, inhibition of adhesion/invasion, antibodies, confocal microscopy, functional assay, single-cell high-content assay

## Abstract

**Introduction:**

*Shigella* is the etiologic agent of a bacillary dysentery known as shigellosis, which causes millions of infections and thousands of deaths worldwide each year due to *Shigella*’s unique lifestyle within intestinal epithelial cells. Cell adhesion/invasion assays have been extensively used not only to identify targets mediating host-pathogen interaction, but also to evaluate the ability of *Shigella*-specific antibodies to reduce virulence. However, these assays are time-consuming and labor-intensive and fail to assess differences at the single-cell level.

**Objectives and methods:**

Here, we developed a simple, fast and high-content method named visual Adhesion/Invasion Inhibition Assay (vAIA) to measure the ability of anti-*Shigella*antibodies to inhibit bacterial adhesion to and invasion of epithelial cells by using the confocal microscope Opera Phenix.

**Results:**

We showed that vAIA performed well with a pooled human serum from subjects challenged with *S. sonnei* and that a specific anti-IpaD monoclonal antibody effectively reduced bacterial virulence in a dose-dependent manner.

**Discussion:**

vAIA can therefore inform on the functionality of polyclonal and monoclonal responses thereby supporting the discovery of pathogenicity mechanisms and the development of candidate vaccines and immunotherapies. Lastly, this assay is very versatile and may be easily applied to other *Shigella* species or serotypes and to different pathogens.

## Introduction

*Shigella* is a Gram-negative bacterium responsible for shigellosis, a severe and often life-threatening gastrointestinal disease (1). Shigellosis is prevalent especially in regions with poor sanitation and overcrowded living conditions, leading to millions of cases and thousands of deaths annually, predominantly among young children (2). Increasing concern is due to the spread of antimicrobial resistance and many outbreaks caused by multi-drug resistant *Shigella* strains have been recently reported both in high and low-middle income countries (3–5). The hallmark of *Shigella* pathogenesis lies in its ability to efficiently invade and colonize the human intestinal epithelial cells, culminating in inflammation, tissue damage, and subsequent clinical symptoms.

*Shigella* pathogenesis involves a sophisticated and multifaceted process that starts with bacterial adhesion to the apical surface of intestinal epithelial cells. This is mediated by putative adhesins and invasins present on the bacterial outer membrane, which interact with specific receptors on the host cell membrane (6). Key invasins include IpaB, IpaC, and IpaD, which contribute to forming the tip of a type III secretion system (T3SS) needle complex responsible for injecting bacterial effectors into the host cell (7). Additionally, the outer membrane protein VirG (IcsA) and its protease IcsP, both essential for actin-based motility, promote bacterial spread within the host cell cytoplasm (8). Following adhesion, *Shigella* triggers a localized actin polymerization response in the host cell, resulting in the formation of membrane ruffles (9), which engulf the bacterium and allow the establishment of its unique intracellular niche (10). Inside the host cell, *Shigella* escapes the phagosomal compartment and resides freely within the host cell cytoplasm (11, 12). Once within the cytoplasm, *Shigella* exploits the host cell actin cytoskeleton to propel itself from one cell to another, facilitating its dissemination throughout the intestinal mucosa (13). *Shigella*-mediated invasion activates various signaling pathways in the host cells, inducing pro-inflammatory responses and the recruitment of immune cells, such as neutrophils and macrophages, to the site of infection (14). This results in the characteristic inflammatory response and the clinical symptoms of shigellosis, including: diarrhea, fever, abdominal pain, and potentially life-threatening complications (1).

The role of antibodies in inhibiting the capacity of *Shigella* to adhere to and invade epithelial cells has been traditionally investigated through colony forming units (CFU)-based assays. During a conventional adhesion/invasion inhibition assay (AIA), *in vitro* cell models of different complexities (from monolayers to differentiated cells) are infected with a defined number of live bacteria opsonized in solution with the antibody of interest (15), followed by removal of non-infecting bacteria, permeabilization of cells and plating of the resulting lysates to determine the number of adherent and internal bacteria by CFU counting. These approaches demonstrated that mouse polyclonal sera and monoclonal antibodies (mAbs) against IpaD (16), IpaB (15, 17) and IpaC (18) were effective in reducing the ability of *Shigella* to infect epithelial cells *in vitro* (19) and confirmed a protective role of anti-Ipa antibodies (20–23). In addition, Caboni et al. utilized the *in vitro* adhesion assay to measure the invasiveness of *Shigella sonnei* strains exhibiting different levels of capsule thickness, demonstrating that the two parameters are inversely related (24). Despite having provided useful insights into *Shigella* pathogenesis, conventional AIA is a labor-intensive, time-consuming and prone to error procedure that fails to assess differences at the single-cell level.

To overcome these limitations, we have developed a high-content microscopy-based assay which, combined with automated single-cell image and statistical analyses, allows rapid and sensitive evaluation of the capacity of anti-*Shigella* antibodies to inhibit bacterial adhesion to and invasion of host cells. We named this new methodology visual AIA (vAIA) and proved its ability to measure the inhibitory activity of human sera derived from subjects challenged with *S. sonnei* (25) and of a specific anti-IpaD mAb to impair *S. sonnei* infection of intestinal epithelial cells.

## Materials and methods

### Cell culture conditions

HT29, CaCo-2 and HeLa cells were purchased from ATCC (US). HT-29 and Caco-2 cells were grown in Dulbecco’s Modified Eagle’s Medium (DMEM), supplemented with GlutaMAX, 4.5 g/L D-Glucose, 1% Pyruvate, 10% Fetal Bovine Serum (FBS), 1% MEM Non-Essential Ammino Acids (NEAA), and in a humidified 5% CO_2_ atmosphere at 37°C. HeLa cells were grown in Eagle’s Minimal Essential Medium (EMEM), supplemented with 10% FBS. All cells were maintained at a concentration of 1x10^6^ cells/mL and passed when 80-90% confluency was reached. All culture media and supplements were obtained from Gibco (Thermo Fisher Scientific, US).

### Bacterial strains growth

*S. sonnei* 53G strains used in this study were grown in Tryptic Soy Broth (TSB) liquid medium or on TSB-agar plates for determination of colony forming units (CFU) at 37°C without CO_2_. Bacteria were first inoculated in 5 ml TSB with the appropriate antibiotics and incubated with shaking at 150 rpm overnight. On the following day, cultures were expanded to 40 ml TSB supplemented with 0.5% bile salts (Sigma Aldrich, Germany) to stimulate the expression of the T3SS (1) and incubated with shaking until bacteria reached the exponential phase of growth (OD_600_ 0.5), which was used to perform all following experiments.

### Generation of mutant *Shigella* strains

Briefly, mutants were generated in *S. sonnei* 53G as follows: stabilization of the virulence plasmid pINV was achieved by insertion of an antibiotic resistance cassette either into the *wbg* cluster (26), responsible for the synthesis of the O-Antigen (OAg- strain), or between *aqpZ* and RS31270 genes (OAg+ strain). Additionally, genes encoding for key components of the T3SS, namely IpaB, IpaD, Spa33, and for the adhesins VirG and IcsP, were deleted by insertion of a kanamycin resistance cassette in the corresponding genes in the OAg- background. The resistance cassette replacement construct was amplified from the pKD4 vector using forward and reverse primers composed of 50 bp homologous to the flanking regions of the gene to be deleted and approximately 20 bp (**Supplementary Table 1**) at the 3′ end, matching the flanking region of the resistance gene. PCR products were purified and used to transform recombination-prone *S. sonnei* recipient cells carrying the temperature-sensitive pAJD434 plasmid. Subsequently, mutants were cured of pAJD434 plasmid by culturing them at 40-42°C. Finally, a strain that spontaneously lost pINV (pINV- strain) was propagated after selection on Congo Red, which is an artificial inducer of *Shigella* T3SS (27). This strain appeared as a white colony on Congo Red agar plates, indicating loss of the virulence plasmid pINV that also encodes the serotype-specific OAg biosynthesis locus in *S. sonnei* (28). **Table 1** lists strains and antibiotics used in this study.

**Table 1:** Bacterial strains and antibiotics used in this study.

Strain name	Description	Antibiotic resistance	Antibiotic concentration
*S. sonnei* 53G OAg+	OAg+	Kanamycin	50 µg/mL
*S. sonnei* 53G OAg-	OAg-	Chloramphenicol	20 µg/mL
*S. sonnei* 53G OAg- IpaB-	OAg- ΔipaB	Chloramphenicol/Kanamycin	20 µg/mL/50 µg/mL
*S. sonnei* 53G OAg- IpaD-	OAg- ΔipaD	Chloramphenicol/Kanamycin	20 µg/mL/50 µg/mL
*S. sonnei* 53G OAg- Spa33-	OAg- Δspa33	Chloramphenicol/Kanamycin	20 µg/mL/50 µg/mL
*S. sonnei* 53G OAg- VirG-	OAg- ΔvirG	Chloramphenicol/Kanamycin	20 µg/mL/50 µg/mL
*S. sonnei* 53G OAg- IcsP-	OAg- ΔicsP	Chloramphenicol/Kanamycin	20 µg/mL/50 µg/mL
*S. sonnei* 53G pINV-	pINV-	None	–

### Generation of fluorescent strains

To visualize and analyze bacteria in vAIA experiments *S. sonnei* 53G strains, described in **Table 1**, were transformed with the integrative plasmid pSEVA23a1 (https://seva-plasmids.com/) containing the gene encoding the fluorescent protein (FP) super folder (sf) mCherry in double copy and in tandem to achieve greater and more consistent expression levels (29). Two tandem copies of the FP were synthesized by the GeneArt company (Thermo Fisher Scientific, US) and provided in the pMK vector. The first copy was codon-optimized for *K. pneumoniae* (https://www.ebi.ac.uk/Tools/st/emboss_backtranseq/), while the second one was specific for *E. coli* to avoid recombination. The synthetic FP gene was subcloned into the HindIII and SpeI restriction sites of the plasmid Multiple Cloning Site (MCS) under control of the pTAC constitutive promoter for efficient expression in Gram-negative bacteria (30). Additionally, a hygromycin resistance cassette was inserted between BamHI and XbaI restriction enzyme sites within the MCS. The presence of the phage phi-C31 integrase and the attP site on the plasmid vector facilitated the integration of the plasmids into the bacterial genome via site-specific recombination with the attB locus on the chromosome (31).

To generate fluorescent bacterial strains, the following workflow was used. First, bacteria were grown until exponential phase. Cells were then centrifuged for 10 min at 5,000 x g and the bacterial pellets were washed 3 times in 20 ml ice-cold 10% glycerol. Finally, electrocompetent cells were resuspended in 1 ml 10% glycerol and 50 μL aliquots were made. 500 ng of plasmid DNA were added to 50 μl of bacteria and transferred to a cold electroporation cuvette. Electroporation was performed by using the Eporator (Eppendorf, Germany) at a voltage of 2500 V and a pulse time of 5-6 ms. Recovery of electroporated bacteria was obtained by adding 500 μl of fresh TSB to the bacterial suspension and incubating at 37°C for 1 h with shaking, before plating onto TSB-agar plates. Characterization of the newly generated fluorescent strains is detailed in **Supplementary Figure 1**.

### Optimization of the visual Adhesion/Invasion Assay

Comparison between conventional AIA and vAIA is shown in **Table 2**. The procedure was optimized using *S. sonnei* 53G OAg- with different cell lines, Multiplicity Of Infections (MOIs) and incubation times. Three different epithelial cell lines, namely CaCo-2, HT-29 and HeLa, were seeded at different concentrations, from 5,000 to 100,000 cells/100 µl, in each well of a flat-bottom 96-well plastic plate (EuroClone, Italy) for conventional AIA, or a 96-well cyclic olefin poly-D-lysine plate (Perkin Elmer, US) for vAIA. Plates were incubated for 2 days at 37°C with 5% CO_2_ to form a monolayer. Cells were then detached using trypsin and counted. *Shigella* strains were grown until exponential phase, centrifuged at 5,000 x g for 10 min and resuspended in DMEM without FBS. Eleven Multiplicity Of Infection ratios (MOIs: MOI5, MOI10, MOI20, MOI30, MOI40, MOI50, MOI60, MOI70, MOI80, MOI90, MOI100) and 6 incubation times (15 min, 30 min, 60 min, 90 min, 120 min, 150 min) were tested (**Supplementary Figure 2**). Fifty microliters of the bacterial suspension, previously incubated for 30 min at 37°C with serial dilutions of the mAbs to be tested, along with appropriate positive (the pooled human serum) and negative (an unrelated mAb against SARS-CoV-2) controls, were added in each well of the plate containing the cells. In parallel, a condition in which the bacteria-mAbs mixture was added to the cells without prior incubation of 30 min at 37°C was also tested (**Supplementary Figure 3**). The plate was then covered with a porous film, centrifuged at 200 x g for 1 min and incubated at 37°C with 5% CO_2_ without shaking for variable amounts of time to allow infection to occur. After infection the bacteria in excess were washed away using DMEM without FBS. From this point on, plates were treated differently.

**Table 2:** Summary of steps for vAIA and comparison with the conventional AIA.

Procedure				Type of assay	
Day	Stage	Step no.	Description	vAIA	Conventional AIA
Day 1	Cell preparation	1	Seed 45000 HT29 cells in 100 µL/well of a 96-well cyclic olefin poly-D-lysine plate (vAIA) or a 96-well plastic plate with flat bottom (conventional AIA)	**√**	**√**
		2	Incubate the plate at 37°C - 5% CO_2_ for 2 days to let the cells form a monolayer	**√**	**√**
Day 2	Bacteria preparation	3	Inoculate sfmCherry-expressing *Shigella* into TSB + appropriate antibiotics	**√**	**√**
		4	Incubate bacteria at 37°C - 150 rpm o/n	**√**	**√**
Day 3		5	Re-launch bacterial culture at 37°C - 150 rpm until exponential phase	**√**	**√**
		6	Resuspend bacteria in DMEM^a^ without FBS^b^	**√**	**√**
	Bacteria opsonization	7	Incubate bacteria (MOI100) with mAb at 37°C for 30 min	**√**	**√**
	Cell infection with pre-opsonized bacteria	8	Discard medium from the original plate	**√**	**√**
		9	Wash the cells 1x with 50 µL/well of DMEM without FBS	**√**	**√**
		10	Add 50 µL/well of bacteria/mAb mix	**√**	**√**
		11	Centrifuge the plate at 200 x g for 1 min	**√**	**√**
		12	Incubate the plate at 37°C - 5% CO_2_ for 1 h	**√**	**√**
		13	Wash the plate 1x with 50 µL/well of DMEM without FBS	**√**	**√**
	Cell permeabilization for CFU determination	14	Permeabilize the cells with 50 µL/well of 1% Triton X-100 (prepared in PBS 1X) at 37°C for 10 min and spot 10 µL of the lysates on TSB^c^-agar plates	**X**	**√**
		15	Incubate the agar plates o/n and count the CFU the day after	**X**	**√**
	Cell fixation	16	Fix the cells with 50 µL/well of 4% PFA^d^ (prepared in PBS 1X) at RT^e^ for 15 min	**√**	**X**
		17	Wash the plate 1x with 50 µL/well of PBS 1X	**√**	**X**
	Staining of bacterial populations	18	Incubate the plate with 50 µL/well of Denka serum (diluted 1:500 in PBS 1X) at RT for 30 min	**√**	**X**
		19	Wash the plate 1x with 50 µL/well of PBS 1X	**√**	**X**
		20	Incubate the plate with 50 µL/well of FITC-conjugated anti-rabbit secondary antibody (diluted 1:2000 in PBS 1X) at RT for 30 min	**√**	**X**
		21	Wash the plate 2x with 50 µL/well of PBS 1X	**√**	**X**
	Counterstaining of bacteria and cells	22	Incubate the plate with 50 µL/well of Cellmask deep red + DAPI (both diluted 1:2000 in PBS 1X) at RT for 10 min	**√**	**X**
		23	Wash the plate 2x with 50 µL/well of PBS 1X	**√**	**X**
	Image acquisition	24	Incubate the plate with 100 µL/well of PBS 1X at 4°C o/n and acquire it at the Opera Phenix the day after	**√**	**X**

a, DMEM, Dulbecco’s Modified Eagle’s Medium. b, FBS, Fetal Bovine Serum. c, TSB, Tryptic Soy Broth. d, PFA, paraformaldehyde. e, RT, room temperature.

In conventional AIA, cells were permeabilized with 1% Triton X-100 (PanReac, AppliChem, US) for 10 min at 37°C and lysates containing both “adherent” and “internal” bacteria were serially diluted, plated as 10 µl spots on TSB-agar plates and incubated at 37°C overnight for CFU determination.

In vAIA, samples were fixed in 4% paraformaldehyde (PFA - Sigma Aldrich, Germany) in PBS 1X for 15 min at room temperature (RT). PFA was then removed, followed by one wash with PBS 1X. Next, plates were incubated for 30 min at RT in 1:500 diluted commercial antiserum (Denka-Seiken, Japan) specific for OAg- *Shigella* strain. One wash in PBS 1X and an additional incubation for 30 min at RT with Alexa Fluor 488 anti-rabbit antibody diluted 1:2,000 in PBS 1X followed. After washing twice with PBS 1X, Cell Mask Deep Red and 4′,6-diamidino-2-phenylindole (DAPI - Thermo Fisher Scientific, US), both diluted 1:2,000, were added for 10 min at RT to stain the cell membrane and the nucleic acids, respectively. Samples were washed and kept in PBS 1X at 4°C overnight before image acquisition. Each sample was tested in triplicate and in at least 2 independent experiments.

### Microscopy and image analysis

Image acquisition was performed by means of the high-content and high-throughput Opera Phenix confocal microscope (Perkin Elmer, US) using the 63X magnification objective, numerical aperture 1.15. At least 21 fields of view and a zoom stack of 5 vertical layers with a distance of 0.5 µm were acquired in each well. The resulting images were analyzed by using the Perkin Helmer Harmony software v. 4.9. Image analysis was performed on the reconstructed maximum projection of the zoom stack and the data represented the average of all fields of view analyzed in each well.

### Visual binding assay

To confirm binding of mAbs to bacteria at single-cell level in the presence of cells, a vBA was performed as follows. First, cells and bacteria were prepared as described in the previous sections. Cells were then infected with 50 µl of the bacterial suspension at MOI100 and the plate was covered with a porous film, centrifuged at 200 x g for 1 min and incubated at 37°C with 5% CO_2,_ without shaking, for 1.5 h. After infection, cells were washed in DMEM without FBS and then fixed in 4% PFA for 15 min at RT. PFA was then removed and cells were further washed in PBS 1X without touching the well bottom. Mild permeabilization of the bacterial membrane in 0.1% of Triton X-100 for 10 min at RT to enhance the accessibility of mAbs to the bacterial antigens was performed (32). After an additional wash in PBS 1X, the mAbs to be tested and appropriate positive and negative controls were added in serial dilutions and the plate was incubated for 30 min at RT. Samples were then washed once with PBS 1X and anti-human or anti-mouse antibodies conjugated to Alexa Fluor 488 (Thermo Fisher Scientific, US) were added at a 1:2,000 dilution for 30 min at RT, followed by two washes with PBS 1X. Finally, the plate was incubated for 10 min at RT in a freshly made solution containing Cell Mask Deep Red and DAPI, both diluted 1:2,000 in PBS 1X. Samples were washed and kept in PBS 1X at 4°C overnight and images were acquired and processed as described in the section above.

### Statistical analyses

All graphs and statistics were generated using GraphPad Prism v. 8.4.3. One-way ANOVA and the unpaired t test were used to compare the infection rates of *Shigella* mutants to those of the parental strain and the effect of the pooled human serum and the anti-IpaD C12-5 mAb to the no-treatment condition. The Pearson r correlation test was used to correlate the results obtained from conventional AIA to the ones of vAIA. All conditions were run in duplicate, and each experiment was repeated at least two times.

### Preparation of the cytosolic and secreted *Shigella* protein fractions

To test mAbs in immunoblot analyses, *Shigella* cytosolic and secreted proteins were obtained using the following procedure. Bacterial strains were grown until exponential phase as previously described. Suspensions were further incubated with 10 µM Congo Red at 37°C without shaking for 30 min and finally pelleted at 5,000 x g for 10 min.

Pellets were weighted and resuspended in a 10-fold volume of lysis buffer from Lipopolysaccharide Isolation Kit (Sigma Aldrich, Germany). Lysates were then filtered (0.22-micron), incubated on ice for 10 min and centrifuged at 16,000 x g for 10 min at 4°C to remove unbroken cells. Aliquots of the resulting supernatants were treated with 0.1 mg/ml proteinase K (Qiagen, Germany) and incubated at 60°C for 1 h, followed by a final step of centrifugation at 16,000 x g for 10 min at 4°C. Supernatants were transferred to a clean tube and quantified by using the Pierce bicinchoninic acid (BCA) protein assay (Thermo Fisher Scientific, US).

Supernatants obtained from the centrifugation of the original cultures were filtered (0.22-micron) and concentrated by centrifugation at 5,000 x g for approximately 30 min using 3 kDa Amicon ultrafiltration devices (Merck Millipore, Ireland). The resulting secretomes were transferred to a clean tube and quantified as described for the lysates.

### Immunoblot analysis

Ten or twenty micrograms of lysates and secretomes were loaded on a NuPAGE Bis-Tris 4-12% protein gel (Thermo Fisher Scientific, US) and run for 1 h at 150 V. The gel was then transferred onto a nitrocellulose membrane with the iBlot2 device (Thermo Fisher Scientific, US) and the membrane was blocked for 2 h at RT in TBST-milk buffer (Tris-Buffered saline (TBS) 1X, containing 0.1% Tween20 and 5% milk). Incubation with 1 µg/ml mAbs prepared in TBST-milk was performed at 4°C rocking overnight. The pooled human serum (diluted 1:1,000 in TBST-milk) and TBST-milk without any mAb were used as a positive and negative control, respectively. The membrane was then washed 3 times with TBST for 3 min each and incubated for 1 h at RT with anti-human or anti-mouse antibodies conjugated to horseradish peroxidase (HRP - Sigma Aldrich, Germany) and diluted 1:75,000 in TBST-milk. A final step of washing with TBST (3 times, 5 min each) followed, before developing the blots by detecting chemiluminescence using the SuperSignal West Pico PLUS Chemiluminescent Substrate (Thermo Fisher Scientific, US) and the iBright 1500 Imagining System (Thermo Fisher Scientific, US).

### Generation of pooled human serum from H03_03TP clinical trial and mAb against IpaD

The pooled human serum utilized as a positive control in this work derives from the sera collected 28 days post-challenge with *S. sonnei* from individuals enrolled in the NCT03527173 clinical trial (25). The study was conducted in accordance with all applicable regulatory requirements, International Conference on Harmonisation - Good Clinical Practice guidelines, and the Declaration of Helsinki. The protocol and study-related documents had been reviewed and approved by the Cincinnati Children’s Hospital Medical Center (CCHMC) institutional review board on 10 May 2018. Written informed consent was obtained from all participants.

The anti-IpaD mAb named C12-5 was obtained from OF1 mice immunized with the purified IpaDHis recombinant protein from *Shigella flexneri* M90T strain (33) and produced according to Guillotte et al. (34). Briefly, 10 µg/dose of the recombinant protein were injected subcutaneously at 3-week intervals. Mice with high specific IgG titers against the immunogen received an intraperitoneal boost immunization 4 days before being sacrificed for splenic B cell fusion. Culture supernatants were first screened by ELISA on the recombinant IpaDHis protein and positive hybridomas were then cloned by limiting dilution and selected based on VarO iRBC surface reactivity by flow cytometry. Monoclonal IgGs were precipitated with 50% ammonium sulfate from ascitic fluid and purified using the Melon™ gel monoclonal IgG purification kit (Thermo Fisher Scientific, US). IgG sub-classes were determined by ELISA using the isotrip mouse mAb isotyping kit (Roche). C12-5 IgG1 was biotinylated using EZ-link sulfo-NHS-biotin (Thermo Fisher Scientific, US), centrifuged through Zeba™ spin desalting columns (Thermo Fisher Scientific, US) and quantified by the Pierce BCA protein assay (Thermo Fisher Scientific, US). This study was carried out in strict accordance with the recommendations in the guide for the care and use of laboratory animals of the Pasteur Institute and complied with the French [ref: 35. Décret n° 2013-118 du 1er février 2013 relatif à la protection des animaux utilisés à des fins scientifiques. NOR: AGRG1231951D] and European Union guidelines for the handling of laboratory animals [ref: Directive 2010/63/EU of the European Parliament and of the Council of 22 September 2010 on the protection of animals used for scientific purposes. Document 32010L0063]. Animal care and handling was approved by the Ministère de l’Agriculture et de la Pêche (Ref 107503056792, issued to OMP) and the protocols and procedures approved by the Direction Départementale des Services Vétérinaires du Préfet de Police de Paris (clearance number C75-273 issued to OMP). All animal experiments were planned and executed to minimize animal suffering.

## Results

### Choice of the cell line to perform vAIA

**Figure 1:**
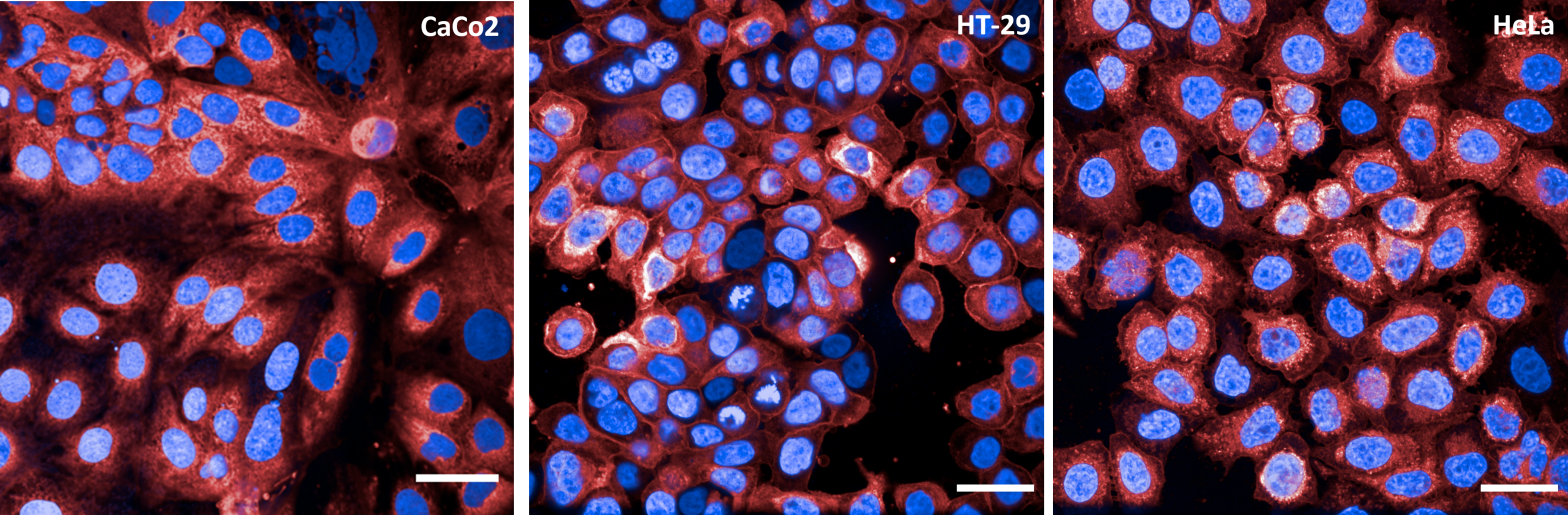
Cell lines tested in vAIA. The panels show representative images corresponding to one field of view acquired for each cell line (CaCo2, HT-29 and HeLa) utilized to establish the assay conditions. Nuclei are in blue and cell membranes in red. All scale bars correspond to 30 µm.

To properly establish a high-content and high-throughput methodology, the choice of the cell line to be used in the assay is pivotal. To this end, 3 different cell lines were analyzed: HeLa, as they are extensively described in the literature for adhesion and invasion assays with Gram-negative bacteria (35–37); CaCo2 and HT29, as they were isolated from intestinal epithelium and are often utilized in adhesion assays for *Shigella* species (38, 39). Each cell line was plated at different densities and incubated at 37°C for 24, 36 and 48 h. At the end of each time point cells were stained with DAPI and Cell Mask Deep Red to visualize nuclei and cell membranes, respectively (**Figure 1**). After microscopy inspection, a cell density of 45,000 cells/well was selected since the desired confluency was reached after 48 h incubation. HT29 was the cell line of choice as these cells displayed a more conventional nuclear state than CaCo2, where polynucleated cells were often observed. In addition, HT29 cells presented the most uniform staining of the cell membrane among the 3 selected cell lines.

### Development of the image analysis pipeline for vAIA

**Figure 2:**
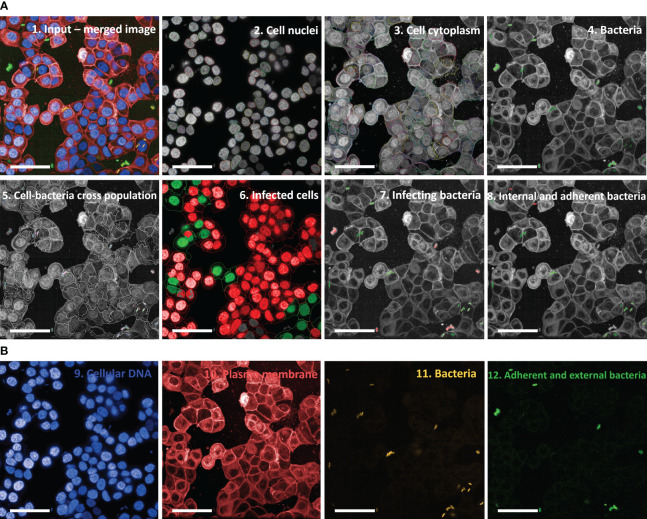
Image analysis pipeline used in vAIA applied to a representative input image. **(A)** Panel 1 depicts a representative input image, which corresponds to the maximum projection of one field of view within a well where bacteria were not treated with any anti-S. sonnei monoclonal antibody. The analysis pipeline developed in this work and applied to the input image is represented following this order: first, cell nuclei are segmented (panel 2), then cell cytoplasm is identified (panel 3). After segmenting bacteria (in green in panel 4), their localization with respect to cells is mapped (panel 5), thus allowing to measure the readouts of the assay: the percentage of infected cells (in green in panel 6), the total number of infecting bacteria (in green in panel 7) and the percentages of adherent and internal bacteria (in red and green in panel 8, respectively). All scale bars correspond to 50 µm. **(B)** The different channels from which the representative image in panel 1 is obtained are represented: nuclei appear blue upon DAPI staining (panel 9), cell membranes are stained red with Cell Mask Deep Red (panel 10) and sfmCherry-expressing bacteria in yellow (panel 11) are further distinguished in external (green and yellow but not in contact with cells), adherent (green and yellow but in contact with cells), and internal (only yellow and associated with cells) (panel 12).

Initial experiments, where HT29 cells were infected with *S. sonnei* and then stained as described in Materials and Methods to identify cells and bacteria, aimed at designing the image analysis pipeline using the Harmony software associated with Opera Phenix. This consisted of a series of consecutive modules (**Figure 2**; **Supplementary Table 2**). Cells were initially identified and counted by segmenting their nuclei after DAPI staining (**Figure 2A**, panel 2 “Cell nuclei” and **Figure 2B**, panel 9 “Cellular DNA”); the corresponding membranes were then localized by staining the cells with Cell Mask Deep Red (**Figure 2A**, panel 3 “Cell cytoplasm” and **Figure 2B**, panel 10 “Plasma membrane”). Next, bacteria were recognized and segmented thanks to the sfmCherry fluorescence signal they expressed (**Figure 2B**, panel 11 “Bacteria”). Expression of sfmCherry is crucial to visualize bacteria, which would not be distinguishable from the cell nuclei based only on DAPI staining (**Figure 2B**, panel 9 “Cellular DNA”). Unspecific signals were excluded based on *Shigella* morphological properties (i.e., area, length, and width). In this module, the objects that the software considers as bacteria are automatically depicted in green, while those excluded from the analysis are depicted in red (**Figure 2A**, panel 4 “Bacteria”). Finally, the number of bacteria and their position with respect to cells was determined by the outcome of the immunostaining. Specifically, bacteria were grouped into 3 different populations: external, adherent and internal bacteria. External bacteria were those bound by the Denka antiserum specific for the OAg- strain but not in contact with the host cells, displaying a green signal decorating the bacterial surface due to the incubation with the Alexa Fluor 488 anti-rabbit antibody (**Figure 2B**, panel 12 “Adherent and external bacteria”). In this module, the software selects only those bacteria that are in contact with the host cells (the infecting bacteria) and depicts them automatically in green, while those excluded from the analysis (the external bacteria) are depicted in red (**Figure 2A**, panel 7 “Infecting bacteria”). Bacteria that exhibited the green signal due to the incubation with the Alexa Fluor 488 anti-rabbit antibody while being near the cells were considered adherent (**Figure 2B**, panel 12 “Adherent and external bacteria”), while those appearing only yellow due to the expression of sfmCherry were considered internal. The immunostaining is crucial to distinguish the adherent from the internal bacteria, considering that the latter could not be reached by the Denka antiserum since cells were not permeabilized in the vAIA procedure. In this module, the software selects only the internal bacteria and depicts them automatically in green, while those excluded from the analysis (the adherent bacteria) are depicted in red (**Figure 2A**, panel 8 “Internal and adherent bacteria”).

Importantly, fluorescence associated with each biological component involved in the experiment was easily detectable when analyzed separately and as a merged image (**Figures 2A, B**). Not only was bacterial and cell fluorescence well defined, but most importantly it was possible to observe a homogenously distributed green signal decorating the surface of adherent and external bacteria. Appropriate negative controls, represented by uninfected cells, were used to evaluate cell autofluorescence in the green channel. Of note, there was no or very low background.

The metrics collected in Harmony were used to quantify the readouts of the assay in terms of both the percentage of infected cells and the total number of infecting bacteria (both depicted in green in the corresponding panels of **Figure 2A**).

### Choice of the bacterial strain to be used in vAIA

*S. sonnei* was chosen for assay establishment and validation because serum samples of individuals who had encountered the pathogen in a controlled human infection model CHIM (25) were available in the laboratory.

**Figure 3:**
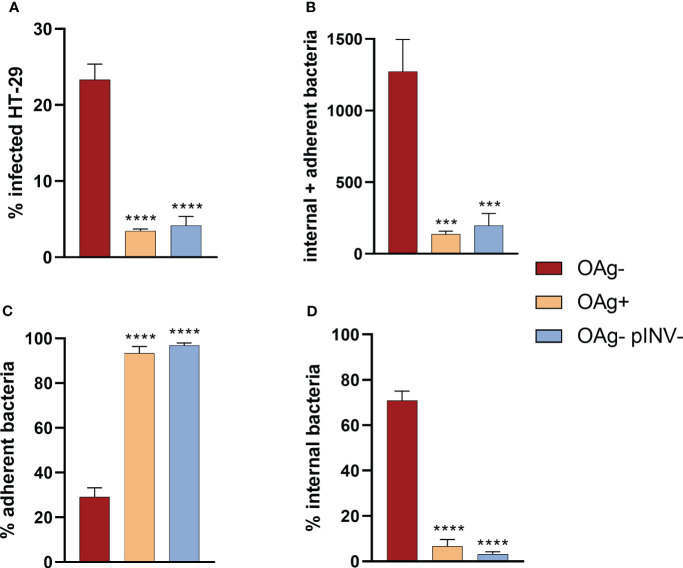
Comparison among the infection rates of *S. sonnei* 53G OAg-, OAg+ and OAg- pINV- strains at MOI 100. The 4 graphs show the differences among these 3 strains in terms of percentage of infected cells **(A)**, absolute number of internal and adherent bacteria **(B)**, and the percentages of adherent **(C)** and internal **(D)** bacteria. One-way ANOVA was used to compare the OAg+ and the OAg- pINV- strains to the OAg- mutant, with significant differences denoted by **** corresponding to a p-value (P) < 0.0001, and *** to P ≤ 0.001.

The infection rates of *S. sonnei* 53G OAg- and OAg+ strains at MOI100 were compared to test whether the absence of the OAg compromised the capacity of bacteria to infect cells, given that the LPS is an important virulence factor of *Shigella* (24), but could also shield virulence proteins. *S. sonnei* 53 OAg+ strain infected HT29 cells approximately 10-fold less than the OAg- mutant (**Figure 3A**, **Supplementary Figure 4**). This statistically significant different infection rate was comparable to that of the non-virulent pINV- strain, which lacks the T3SS (**Figure 3B**, **Supplementary Figure 4**). The same trend was observed for the total number of infecting bacteria (internal + adherent bacteria) and when considering separately the percentages of adherent and internal bacteria (**Figures 3C, D**, **Supplementary Figure 4**). Results obtained at MOI30 (see **Supplementary Figure 5**) reflected what was seen at MOI100, even though differences between strains were less pronounced. Taken together, these observations confirmed the importance of T3SS for the virulence of *Shigella* and the masking effect that the LPS has on the T3SS in *S. sonnei*, which has been already reported in the literature and is further discussed in the Discussion section. As we were interested in maximizing the ability of vAIA to detect different infection rates after treatment with sera and mAbs, we decided to focus on the OAg- strain, since it infected HT29 cells more proficiently.

### Assessing the infection rates of mutant *Shigella* strains

To further evaluate the sensitivity of vAIA in discriminating the adhesion and virulence properties of different *S. sonnei* OAg- strains, mutants in single components of the T3SS (IpaB, IpaD and Spa33) and in the 2 proteins involved in *Shigella* adhesion and invasion of host cells (VirG and IcsP) were assessed.

**Figure 4:**
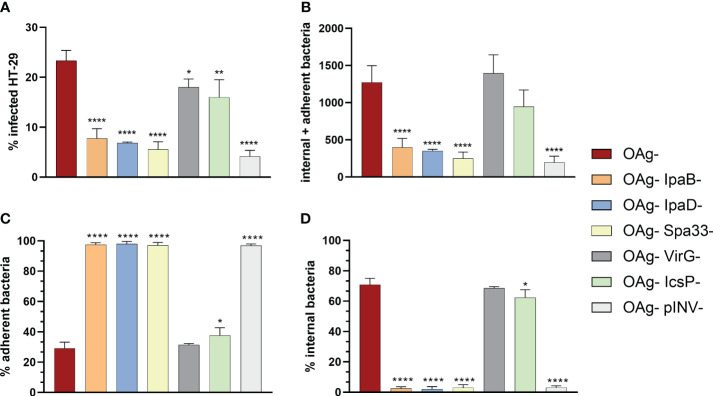
Comparison among the infection rates of *S. sonnei* 53G OAg-, the isogenic mutants in *ipaB*, *ipaD*, *spa33*, *virG*, *icsP* and OAg- pINV- strains at MOI 100. The graphs show the differences calculated using one-way ANOVA in terms of percentage of infected cells **(A)**, absolute number of internal and adherent bacteria **(B)**, and percentages of adherent **(C)** and internal **(D)** bacteria, between the various mutants and the parental OAg- strain. Statistically significant differences are denoted by **** corresponding to P<0.0001, ** to P ≤ 0.01 and * to P ≤ 0.05.

A statistically significant difference between the infection rate of *S. sonnei* 53G OAg- and the corresponding mutants was observed (**Figure 4A**). The percentage of infected cells for the control strain (OAg-) was almost 5-fold higher compared to that of OAg- IpaB-, OAg- IpaD- and OAg-Spa33- mutant strains. The difference between the OAg- strain and the OAg- VirG- and OAg- IcsP- mutants was less pronounced. These results were mirrored by the total number of infecting bacteria (internal + adherent bacteria – **Figure 4B**). Interestingly, the behavior of the non-virulent pINV- strain was similar to that of the OAg- IpaB-, OAg- IpaD- and OAg- Spa33- strains, presenting approximately 4% of infected cells, thus confirming the important role played by IpaB, IpaD and Spa33 in *Shigella* pathogenesis. When considering separately the percentage of adherent and internal bacteria, no internal bacteria were counted for mutants of IpaB, IpaD, Spa33 and the whole pINV when compared to the OAg- control strain (**Figures 4C, D**). In the case of OAg- VirG- and OAg- IcsP- strains, the percentage of adherent bacteria was comparable to the OAg- strain, as well as the percentage of internal bacteria (**Figures 4C, D**), suggesting that the roles of VirG and IcsP might be less critical in the *in vitro* conditions tested.

Overall, these data suggested that the visual assay could be exploited for evaluating the reduction in bacterial adhesion/invasion caused by genetic or functional inactivation of virulence factors.

### Evaluation of the effect of a pooled human serum on *Shigella* infection

After defining the best conditions for vAIA, the capacity of a pooled human serum derived from subjects challenged with *S. sonnei* in a CHIM (25) to inhibit adhesion to and/or invasion of epithelial cells was tested. Comparison with the standard assay, represented by the conventional AIA, was used to validate results.

**Figure 5:**
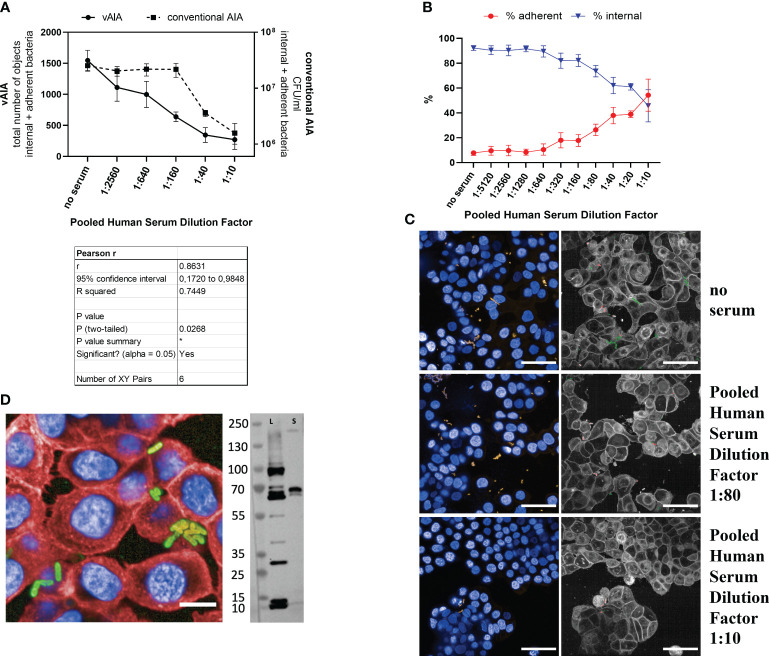
Evaluation of the effect of a pooled human serum on *Shigella* infection. **(A)** The graph illustrates the relationship between the pooled human serum dilution factor and the total number of objects corresponding to the internal and adherent bacteria in vAIA as identified by the Harmony software and the CFU/ml values corresponding to the internal and adherent bacteria as measured by conventional AIA when infecting HT-29 cells with *S. sonnei* 53G OAg- strain. The Pearson r test represented in the table demonstrates a statistically significant correlation between the two assays. **(B)** The graph depicts the percentage of adherent and internal bacteria analyzed separately in vAIA. Pooled human serum dilution factors are indicated on the X-axis. **(C)** The images represent *S. sonnei* 53G OAg- infecting HT-29 cells without adding any serum (“no serum” label) or upon addition of two increasing concentrations of pooled human serum. Nuclei appear blue due to DAPI staining, sfmCherry-expressing bacteria in yellow. In the corresponding segmented panels, internal bacteria are identified in green while adherent bacteria in red. Scale bars correspond to 50 µm. **(D)** In the image, the binding of the pooled human serum at the highest concentration used (dilution factor 1:10) is depicted as a homogenously distributed green signal around the bacterial surface due to the incubation with the Alexa Fluor 488 anti-human secondary antibody. Bacteria express the sfmCherry fluorescent protein in their cytosol, while cell nuclei and membranes are stained with DAPI and Cell Mask Deep Red, respectively. The scale bar corresponds to 10 µm. The blot represents the binding of the serum to *S. sonnei* 53G OAg- lysate (L) and secretome (S). *, P ≤ 0.05.

The outcomes of both vAIA and conventional AIA showed that the absolute number of internal and adherent *S. sonnei* 53G OAg- bacteria significantly decreased in a dose-depended manner with the increasing concentration of the pooled human serum (**Figure 5A**). Pearson r test confirmed a statistically significant correlation between the two procedures (r=0.8631; P<0.05), thus highlighting the accuracy of the newly developed protocol in reproducing results obtained using the standard assay. Moreover, in both assays it was possible to observe a statistically significant reduction (P<0.05) in the total number of internal and adherent bacteria upon comparing the serum at the highest concentration used with the no-serum condition. By looking at the relative percentages of adherent and internal bacteria, a significant 40% increase in the number of adherent bacteria was noted, together with a 46.5% decrease of the internal ones (**Figure 5B**).

Images in **Figure 5C** exemplify the above: in the wells where no serum was added the overall number of bacteria was higher compared to those wells where samples were treated with increasing concentrations of serum. Furthermore, in the no-serum condition most bacteria were internal (depicted in green in the segmented panels), while upon addition of serum almost all bacteria were located outside the host cells (in red in the segmented panels). A homogenous binding of the serum around the bacterial surface was observed at the highest dose used (dilution factor 1:10) in a vBA assay (**Figure 5D**). Immunoblot analysis further confirmed binding of the pooled human serum to different antigens of the *S. sonnei* 53G OAg- total protein extract (**Figure 5D**), with an observed enrichment of the binding signal to proteins at around 70 kDa mass size in both the whole lysate and the secretome of bacteria. Taken together, these data indicate that a polyclonal human serum can inhibit invasion of HT-29 cells by the *S. sonnei* 53G OAg- strain.

### Application of vAIA to the assessment of mAb functionality

Given the promising results obtained with the pooled human serum, vAIA was employed to evaluate the efficacy of a specific mAb against a key component of the T3SS in inhibiting the invasion of *Shigella*. To do so, the murine mAb targeting IpaD, named C12-5, was tested.

**Figure 6:**
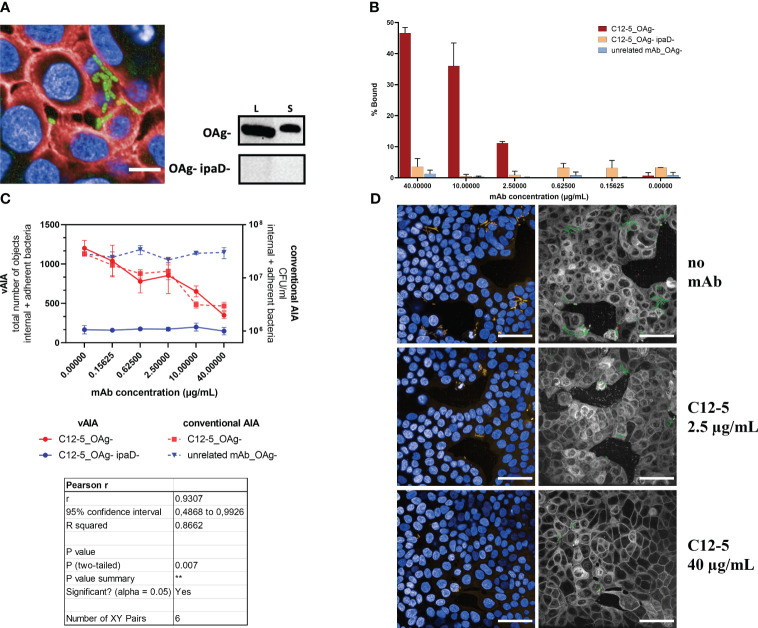
Evaluation of the effect of an anti-IpaD mAb on *Shigella* infection. **(A)** In the image, the binding of C12-5 at the highest concentration used (40 µg/mL) is depicted as a homogenously distributed green signal around the bacterial surface due to the incubation with the Alexa Fluor 488 anti-mouse secondary antibody. Bacteria express the sfmCherry fluorescent protein in their cytosol, while cell nuclei and membranes are stained with DAPI and Cell Mask Deep Red, respectively. The scale bar corresponds to 10 µm. The blots represent the binding of C12-5 to *S. sonnei* 53G OAg- and OAg- ipaD- lysates (L) and secretomes (S). **(B)** The graph shows the vBA results obtained upon testing increasing concentrations of C12-5 against the OAg- strain. Negative controls are represented by the OAg- ipaD- strain and by an unrelated mAb against SARS-CoV-2 tested against the OAg- strain. **(C)** The graph illustrates the relationship between the anti-IpaD C12-5 mAb concentration and the total number of objects corresponding to the internal and adherent bacteria in vAIA as identified by the Harmony software and the CFU/ml values corresponding to the internal and adherent bacteria as measured by conventional AIA when infecting HT-29 cells with *S. sonnei* 53G OAg- strain. Negative controls were represented by the OAg- ipaD- strain and by an unrelated mAb against SARS-CoV-2 for vAIA and conventional AIA, respectively. The Pearson r test represented in the table highlights a statistically significant correlation between the two assays. **(D)** The images represent *S. sonnei* 53G OAg- strain infecting HT-29 cells without adding any mAb and upon addition of two increasing concentrations of C12-5. Nuclei appear blue due to DAPI staining, sfmCherry-expressing bacteria in yellow. In the corresponding segmented panels, the total number of infecting bacteria is depicted in green. Scale bars correspond to 50 µm. **, P ≤ 0.01.

The C12-5 anti-IpaD mAb was able to bind homogeneously *S. sonnei* 53G OAg- in vBA (**Figure 6A**) and immunoblot analysis revealed that C12-5 identified a protein of 40 kDa in both lysate and secretome of *S. sonnei* 53G OAg- (**Figure 6A**). No signal was detected in the lysate and secretome preparation from the negative control strain lacking IpaD (**Figure 6A**), thus confirming its specificity. Image analysis of vBA showed more than 40% of bound bacteria at the highest dose (40 µg/mL) of C12-5, whilst the percentage decreased in a dose-dependent manner with the decreasing concentration of the mAb (**Figure 6B**). No, or very low, binding was detected in the negative controls (C12-5 against the IpaD-deficient strain and by using an unrelated mAb against SARS-CoV-2). When tested in vAIA and conventional AIA, C12-5 reduced the total number of internal and adherent bacteria in a dose-dependent fashion (**Figure 6C**), indicating that C12-5 inhibited invasion of *S. sonnei* 53G OAg- strain in HT-29 cells. Pearson r test confirmed a statistically significant correlation between the two procedures (r=0.9307; P<0.01), thus highlighting the accuracy of the newly developed protocol in reproducing results obtained using the standard assay also with a mAb. Additionally, a statistically significant reduction (P<0.05) in the total number of internal and adherent bacteria was measured in both assays upon comparing the mAb at the highest concentration used with the no-mAb condition. No effect was observed on the mutant *S. sonnei* OAg- lacking IpaD and with an unrelated mAb against SARS-CoV-2, used as negative controls. Images in **Figure 6D** are representative of the above: in the wells where no mAb was added the overall number of infecting bacteria (in green in the segmented panels) was higher compared to those wells where samples were treated with increasing concentrations of C12-5. Notably, at the highest mAb dose (40 µg/mL) the number of internal and adherent bacteria measured in vAIA (**Figure 6C**) was almost the same as that of the negative control used in the assay (OAg- IpaD-), suggesting that C12-5 effectively neutralized *S. sonnei* 53G OAg- ability to invade host cells.

## Discussion

In this study, a method called vAIA for high-content microscopy-based screening of mAbs capacity to inhibit adhesion and invasion of *S. sonnei* into intestinal epithelial cells was developed, thereby reducing experimental time and cost. In addition, being operated by Opera Phenix, this assay can be optimized in high-throughput format. To ensure that the assay conditions were appropriate and sensitive for measuring the inhibitory effects of mAbs on pathogen attachment and invasion mechanisms, key parameters such as MOI and incubation time were optimized. This led to the choice of MOI100, which represented the situation in which almost all bacteria were internal, and 1 h incubation time, which was characterized by the highest percentage of adherent bacteria and by the lowest percentage of internal ones (see **Supplementary Figure 2**). The assay was then established by using mutants in key components of the T3SS, which displayed a significantly reduced virulence compared to the parental strain. Next, vAIA demonstrated that a pooled human serum derived from subjects challenged with *S. sonnei* significantly affected the capacity of *S. sonnei* to infect HT-29 cells. The use of this reagent was key to the setup of the assay and to ensure that results obtained were biologically translatable to human pathogenicity. Finally, the newly developed procedure was successfully applied to a dose-dependent assessment of an anti-IpaD mAb, which can be used as a positive control to evaluate the functionality of anti-*Shigella* mAbs.

Evaluating the infection rates of various *Shigella* strains is crucial not only to comprehend their virulence and pathogenic potential, but also because it aids in understanding their distribution, frequency, and modes of transmission. This knowledge will help in devising efficient strategies for outbreak prevention, control, and proper treatment selection (40–42). In this study, the evaluation mainly focused on strains belonging to *S. sonnei* 53G genotype, being one of the two main pathogenic strains of *Shigella* species (*S. sonnei* and *S. flexneri* (12)) and for which literature is limited. When the infection rate of the *S. sonnei* OAg+ strain was compared to that of the OAg- counterpart, vAIA revealed that the former infected the cells approximately 10-fold less efficiently than the OAg- mutant, an observation already made by using conventional AIA. Caboni et al., for example, found that accessibility of IpaB increased upon removal of the G4C capsule, and a further increase was observed for an OAg-deficient strain, indicating that the OAg contributes to shielding the T3SS and, consequently, *Shigella* virulence (24). Watson and collaborators demonstrated, using the standard assay, that efficient macrophage invasion by *S. sonnei* is only possible when the OAg layer and the G4C capsule are removed (9). When dissecting the contribution of key components of the T3SS to the invasiveness of *S. sonnei*, vAIA demonstrated that the percentage of infected cells and the total number of infecting bacteria for the OAg- strain was significantly higher compared to that of OAg- IpaB-, OAg- IpaD- and OAg- Spa33- mutans, while this difference was less pronounced for strains lacking VirG and IcsP. This could be explained by the fact that IpaB, IpaD, and Spa33 proteins are part of the T3SS and implicated in the initial contact with host cells (43). Conversely, VirG and IcsP function as adhesins that cooperate with the T3SS to invade the host cells (44, 45), suggesting that their roles might be less critical. These results are in accordance with those found by High and collaborators, who demonstrated that a *Shigella* mutant strain that does not express IpaB but expresses downstream genes is non-invasive, does not elicit actin polymerization, but can bind to HeLa cells (46). Similarly, Schiavolin et al. have seen that *Shigella* mutants for IpaD are less invasive compared to the parental strain (47). Schuch and coworkers’ study has also demonstrated that a Spa33-null mutant was both noninvasive and unable to translocate the Ipa proteins from the inner to the outer membrane of the Mxi-Spa transmembrane channel (48). Taken together, the results of this work confirmed the observations previously made with standard assays, indicating that the newly developed visual methodology is reliable and robust.

Importantly, the inhibition curves obtained with the pooled human serum and a mAb with known target both statistically correlated to those derived by using the standard procedure. The dose-dependent inhibition curve observed for the anti-IpaD C12-5 mAb highlights the importance of this protein in *Shigella* pathogenesis (7). IpaD represents a crucial component of the T3SS needle complex, serving as a scaffold protein for IpaB and as a signal transducer to activate effector secretion (7). IpaB is located at the needle tip with IpaD and together they form the hydrophobic translocon, which delivers effectors to host cell membranes (49). IpaD is also involved in the cytoplasmic signal transduction pathway, working alongside MxiC to fully activate the T3SS and secrete the remaining effectors (50). Although the initial sensing of the host cell membrane by IpaB is not yet fully understood, it is known that IpaD plays a critical role in co-transducing this signal down the T3SS needle to activate secretion (51, 52), and this could explain the effect that was measured in vAIA. The response obtained with the anti-IpaD mAb highlights the potential of vAIA to identify antibodies that are usually overlooked during conventional bactericidal assays. Given the automated single-cell image analysis, vAIA has the potential to replace standard infection assays in upcoming research, especially in high-sensitive contexts.

In conclusion, the innovative vAIA methodology can be exploited in the search for inhibitors of *S. sonnei* adhesion to and invasion of epithelial cells. By providing an efficient system for assessing the functionality of sera and mAbs with known target, this approach can facilitate high-throughput evaluation of anti-bacterial mAbs that inhibit invasiveness of *Shigella* and guide the discovery of previously unknown surface antigens, thus paving the way for more effective treatments. Such innovative tools are essential to advance our understanding and to develop new therapeutic strategies, which is crucial in the fight against antimicrobial resistant pathogens.

## Data availability statement

The raw data supporting the conclusions of this article will be made available by the authors, without undue reservation.

## Ethics statement

The studies involving humans were approved by Cincinnati Children’s Hospital Medical Center (CCHMC) institutional review board on 10 May 2018. The studies were conducted in accordance with the local legislation and institutional requirements. The participants provided their written informed consent to participate in this study. The animal study was approved by Direction Départementale des Services Vétérinaires du Préfet de Police de Paris (clearance number C75-273 issued to OMP). The study was conducted in accordance with the local legislation and institutional requirements.

## Author contributions

GB: Conceptualization, Formal analysis, Investigation, Methodology, Visualization, Writing – original draft, Writing – review & editing. GV: Conceptualization, Formal analysis, Investigation, Methodology, Visualization, Writing – original draft, Writing – review & editing. SL: Investigation, Methodology, Writing – original draft. AA: Conceptualization, Investigation, Resources, Writing – review & editing. DC: Data curation, Investigation, Methodology, Writing – original draft. MR: Investigation, Methodology, Writing – review & editing. EB: Investigation, Methodology, Writing – review & editing. ER: Methodology, Writing – review & editing. MR: Methodology, Writing – review & editing. GG: Methodology, Writing – review & editing. MP: Supervision, Writing – review & editing. OR: Supervision, Writing – review & editing. FBS: Funding acquisition, Supervision, Writing – review & editing. FM: Conceptualization, Funding acquisition, Supervision, Writing – review & editing. RR: Funding acquisition, Supervision, Writing – review & editing. CS: Conceptualization, Funding acquisition, Project administration, Supervision, Validation, Writing – original draft, Writing – review & editing.
