# Supplementary material for: Development of a visual Adhesion/Invasion Inhibition Assay to assess the functionality of Shigella-specific antibodies
Source: Front Immunol. 2024 Apr 12;15:1374293. doi: 10.3389/fimmu.2024.1374293 (PMC11045934; doi:10.3389/fimmu.2024.1374293)
Supplement: Supplementary file 1 [file DataSheet_1.pdf]

Supplementary material for

Development of a visual Adhesion/Invasion Inhibition Assay to assess the functionality of *Shigella*-specific antibodies

G. Batani[1,7], G. Vezzani[2,7], S. Lashchuk[1], A. Allaoui[3], D. Cardamone[4], MM. Raso[2], E. Boero[2], E. Roscioli[1], M. Ridelfi[1], G. Gasperini[2], M. Pizza[5], O. Rossi[2], F. Berlanda Scorza[2], F. Micoli[2], R. Rappuoli[6] and C. Sala[1*]

[1] Monoclonal Antibody Discovery Laboratory, Fondazione Toscana Life Sciences, Siena, Italy

[2] GSK Vaccines Institute for Global Health (GVGH), Siena, Italy

[3] University Mohammed VI Polytechnic, Ben Guerir, Morocco

[4] Data Science for Health Laboratory, Fondazione Toscana Life Sciences, Siena, Italy

[5] Department of Life Sciences, Imperial college, London, United Kingdom

[6] Fondazione Biotecnopolo di Siena, Siena, Italy

[7] These authors contributed equally

*Correspondence: c.sala@toscanalifesciences.org

Supplementary Table 1. List of primers used in this study.

**Primer name**	**Sequence (5'→3')**
IpaB KO F	GGCTTCCACTGAGCTTGGAGACAATACTATCCAGGCTGCAAATGATGC AGGTGTAGGCTGGAGCTGCTTC
IpaB KO R	CCTGAGAGTTGGACATTGAGGCTAATAGATCTGCAATTACTTCCTGCA ATCATATGAATATCCTCCTTAG
IpaD KO F	AAACAATACCAACGGTTCATCAACCGAAACAGTTAATTCTGATATAAA AAGTGTAGGCTGGAGCTGCTTC
IpaD KO R	TACTCAAAACCTTTACTAAATTATCAAAAATACTATTGGCATTACTGTA TCATATGAATATCCTCCTTAG
Spa33 KO F	AAAATACGAGGTATTTTCAGGATTTTCTACACAAGAATCTTTACTGCAT TGTGTAGGCTGGAGCTGCTTC
Spa33 KO R	TAGAAACAAGTTCCCCATGCCCAACCAAAGAACCATTTACTTTAATAT TTCATATGAATATCCTCCTTAG
IcsP KO F	TACACATGCTACCACTAACTATCCACTTTTCATACCGGACAACATCAG TAGTGTAGGCTGGAGCTGCTTC
IcsP KO R	TACTTGCACTATTTTTAATGGTGCCAGATATATTGGTACTTTGCTCATAA CATATGAATATCCTCCTTAG
VirG KO F	ATGAATCAAATTCACAAATTTTTTTGTAATATGACCCAATGTTCACAGG GGTGTAGGCTGGAGCTGCTTC
VirG KO R	TCAGAAGGTATATTTCACACCCAAAATACCTTGGGTGTCTCTGTAACT GTCATATGAATATCCTCCTTAG

Supplementary Table 2. Image analysis pipeline developed in this work using the Perkin Helmer Harmony software v. 4.9.

	Input	Method	Output
**Input Image**	Flatfield Correction: Basic<br>Stack Processing: Maximum Projection<br>Min. Global Binning: Dynamic		
**Find Nuclei**	Channel: DAPI<br>ROI: None	Method: B<br>Common Threshold: 0.65<br>Area > 20 µm²<br>Splitting Coefficient: 100<br>Individual Threshold: 0.45<br>Contrast > 0.1	Output Population: HT29
**Find Cytoplasm**	Channel: CellMask Deep Red<br>Nuclei: HT29	Method: D<br>Individual Threshold: 0.3	
**Select Population**	Population: HT29	Method: Common Filters<br>Region: Cell	Output Population: HT29 Selected for analysis
**Find Spots**	Channel: mCherry<br>ROI: None	Method: D<br>Detection Sensitivity: 0.2<br>Splitting Sensitivity: 0.55<br>Background Correction: 0.5<br>Calculate Spot Properties	Output Population: bacteria
**Select Population (2)**	Population: bacteria	Method: Common Filters<br>Remove Border Objects<br>Region: Spot	Output Population: bacteria filtered

	Input	Method	Output
**Calculate Morphology Properties**	Population bacteria filtered<br>Region: Spot	Method: Standard<br>Area<br>Width<br>Length	Property Prefix: Morphology Shigella
**Select Population (3)**	Population: bacteria filtered	Method: Filter by Property<br>Morphology Shigella Area [µm²]: > = 1<br>Morphology Shigella Width [µm]: > 0.5<br>Morphology Shigella Length [µm]: > 1<br>Boolean Operations: F1 and F2 and F3	Output Population: bacteria selected for analysis by morphology
**Select Region**	Population: bacteria filtered<br>Region: Spot	Method: Resize Region [µm / px]<br>Outer Border: -0.2 µm<br>Restrictive Population: None<br>Restrictive Region:<br>Inner Border: INF µm	Output Region: Bacteria Resized
**Calculate Position Properties**	Population: bacteria filtered<br>Region: Bacteria Resized	Method: Cross Population<br>Population B: HT29<br>Region B: Cell<br>ROI Border Distance<br>Overlap	Property Prefix: bacteria position to HT29 for analysis
**Select Population (3)**	Population bacteria filtered	Method: Filter by Property	Output Population: Internal + Adherent bacterial

		bacteria<br>position to<br>HT29 for<br>analysis<br>Overlap [%]:<br>> 0	
**Select Population (4)**	**Input**	**Method**	**Output**
	Population bacteria filtered	Method:<br>Filter by<br>Property<br>bacteria<br>position to<br>HT29 for<br>analysis<br>Overlap [%]:<br>< = 0	Output Population: External bacteria
**Calculate Intensity Properties**	**Input**	**Method**	**Output**
	Channel: CellMask Deep Red<br>Population Internal + Adherent bacteria<br>Region: Bacteria Resized	Method:<br>Standard<br>Mean	Property Prefix: Intensity Bacteria Resized CellMask Deep Red
**Select Population (5)**	**Input**	**Method**	**Output**
	Population Internal + Adherent bacterial	Method:<br>Filter by<br>Property<br>Intensity<br>Bacteria<br>Resized<br>CellMask<br>Deep Red<br>Mean: > 500	Output Population: Adherent bacteria Selected
**Select Population (6)**	**Input**	**Method**	**Output**
	Population Internal + Adherent bacterial	Method:<br>Select by<br>Mask<br>Region: Spot<br>Mask<br>Population:<br>Adherent<br>bacteria<br>Selected<br>Mask<br>Region: Spot<br>Select by<br>Overlap > 50<br>%<br>Use Inverted<br>Mask	Output Population: Internal bacteria Selected
**Calculate Position Properties (2)**	**Input**	**Method**	**Output**
	Population: HT29<br>Region: Cell	Method:<br>Cross<br>Population<br>Population<br>B: Internal +<br>Adherent<br>bacteria<br>Region B:<br>Spot<br>ROI Border	Property Prefix: HT29 position to Internal + Adherent bacteria

		Distance<br>Overlap	
**Select Population (7)**	**Input**	**Method**	**Output**
	Population: HT29	Method: Filter by Property<br>HT29 position to Internal + Adherent bacteria Overlap [%]: > 0	Output Population: Infected HT29 (internal + adherent)
**Select Population (8)**	**Input**	**Method**	**Output**
	Population: HT29	Method: Select by Mask<br>Region Cell<br>Mask Population: Infected HT29 (internal + adherent)<br>Mask Region Cell<br>Select by Overlap > 50 %<br>Use Inverted Mask	Output Population: Not infected HT29
**Define Results**	**Results**		
	**Method**: Standard Output<br>Internal + Adherent bacteria - Number of Objects: Object Count<br>Output Name: Internal + Adherent bacteria - Number of Objects<br><br>**Method**: Formula Output<br>Formula: i/k*100<br>Population Type: Objects<br>Variable i: Internal + Adherent bacteria - Number of Objects<br>Variable k: bacteria filtered - Number of Objects<br>Output Name: % Internal+Adherent<br><br>**Method**: Formula Output<br>Formula: e/k*100<br>Population Type: Objects<br>Variable e: External bacteria - Corrected Spot Intensity Mean<br>Variable k: bacteria filtered - Number of Objects<br>Output Name: % External<br><br>**Method**: Formula Output		

Formula: i/Y
Population Type: Objects
Variable i: Internal + Adherent bacteria - Number of Objects
Variable Y: Infected HT29 (internal+adherent) - Number of Objects
Output Name: Mean N Infecting per infected HT29

Method: Formula Output
Formula: a/i*100
Population Type: Objects
Variable a: Adherent bacteria Selected - Number of Objects
Variable i: Internal + Adherent bacteria - Number of Objects
Output Name: % adherent bacteria

Method: Formula Output
Formula: c/i*100
Population Type: Objects
Variable c: Internal bacteria Selected - Number of Objects
Variable i: Internal + Adherent bacteria - Number of Objects
Output Name: % internal bacteria

Method: Formula Output
Formula: a/b*100
Population Type: Objects
Variable a: Infected HT29 (internal+adherent) - Number of Objects
Variable b: HT29 Selected for analysis - Number of Objects
Output Name: % infected HT29

Method: Standard Output
Adherent bacteria Selected - Number of Objects: Object Count
Output Name: Adherent bacteria Selected - Number of Objects

Method: Standard Output
Internal bacteria Selected - Number of Objects: Object Count
Output Name: Internal bacteria Selected - Number of Objects

Object Results
Population: Internal + Adherent bacteria: ALL
Population: bacteria filtered: ALL
Population: bacteria: ALL
Population: bacteria selected for analysis by morphology: ALL
Population: External bacteria: ALL
Population: Adherent bacteria Selected: ALL
Population: Internal bacteria Selected: ALL
Population: HT29: ALL
Population: HT29 Selected for analysis: ALL
Population: Infected HT29 (internal+adherent): ALL
Population: Not infected HT29: ALL

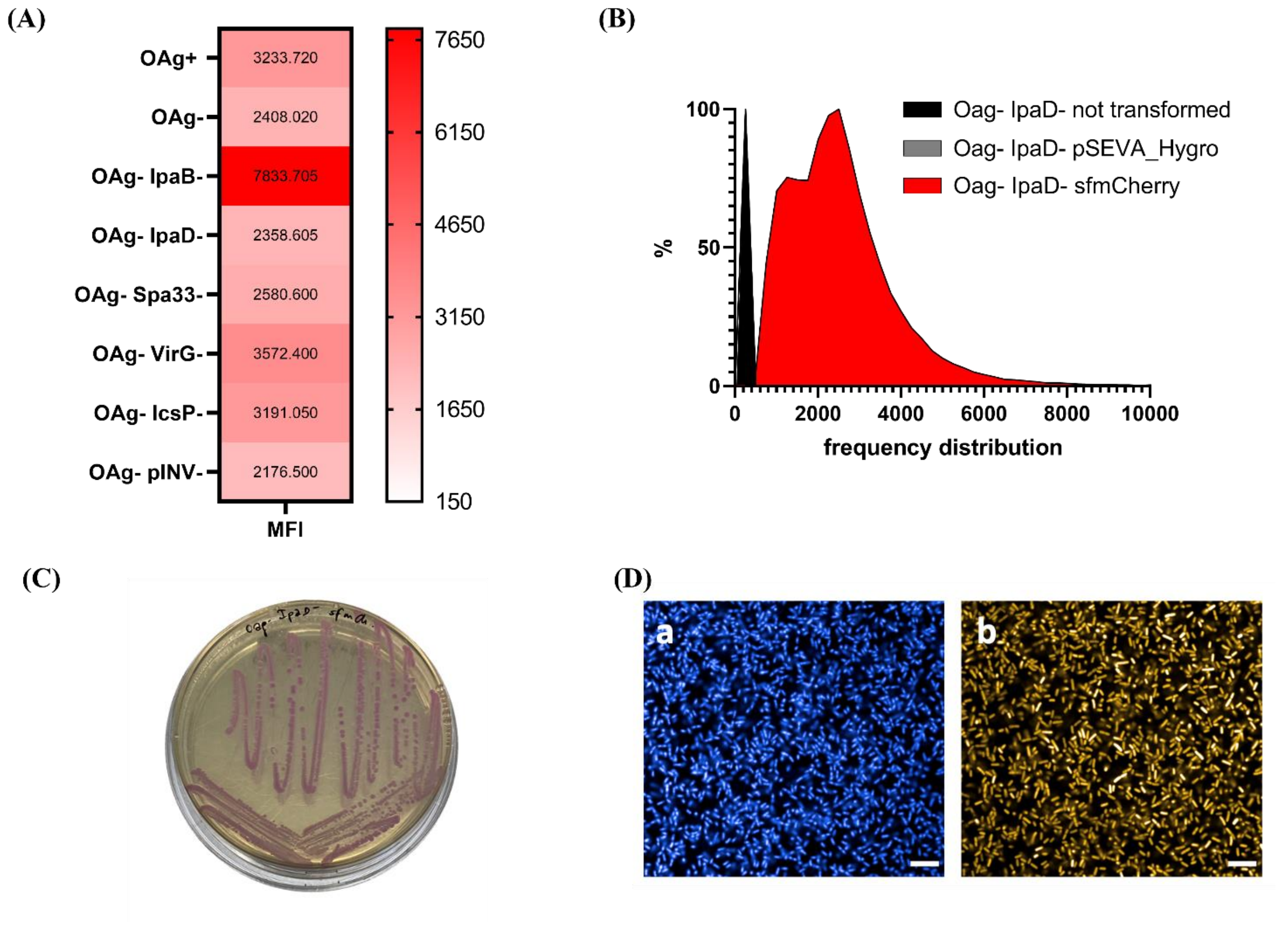

Supplementary Figure 1. Results of quantitative image analysis by Opera Phenix confirmed successful generation of fluorescent strains. **(A)** The heat map represents the MFI of *Shigella* strains carrying the gene encoding for sfmCherry. MFI values are coloured by shades of red depending on the relative intensity of each strain. **(B)** The graph depicts an example of frequency distribution, on X axis, of signal intensity, on Y axis, for *S. sonnei* OAg- IpaD- carrying the gene encoding sfmCherry when compared to the negative controls represented by the parental strain (OAg- IpaD- not transformed) and *S. sonnei* OAg- IpaD- transformed with the pSEVA plasmid without the gene encoding for sfmCherry (OAg- IpaD- pSEVA_Hygro). **(C)** SfmCherry colonies appearing pink on TSB-agar plates are also shown. **(D)** Panel a depicts DAPI staining of the representative strain, while panel b illustrates the corresponding bacteria expressing sfmCherry. In both images, scale bars correspond to 20 µm.

(A)

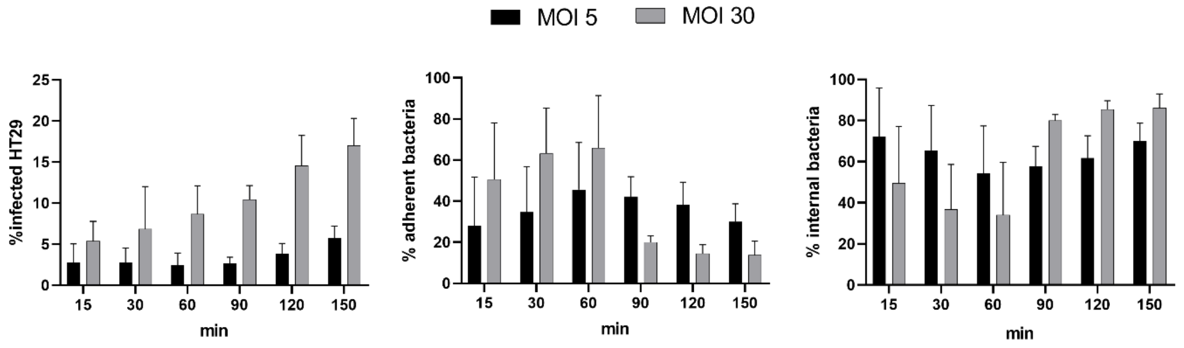

(B)

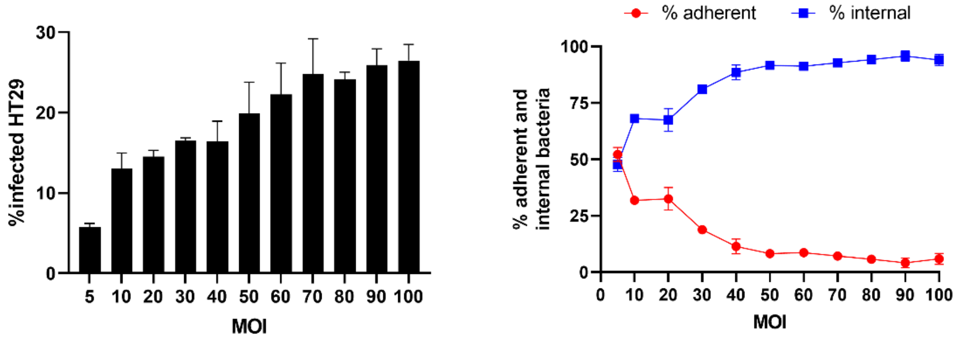

Supplementary Figure 2. Results of optimization of the incubation time and the MOI used in vAIA. **(A)** The three graphs depict the variations in the percentage of infected HT-29 cells with *S. sonnei* OAg- strain, in the percentage of adherent and internal bacteria, on the Y axes, over a time lapse ranging from 15 to 150 min, on the X axes, at MOI 5 and MOI 30. **(B)** The graphs illustrate the relationship between the MOI, on the X axis, and the percentage of infected HT-29 cells, on the Y axis on the graph on the left, and that of adherent and internal bacteria, on the Y axis on the graph on the right.

(A)

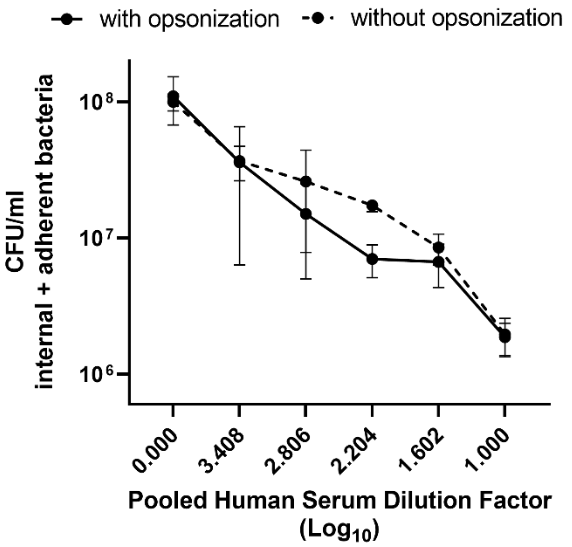

(B)

**Pearson r**	
r	0.9905
95% confidence interval	0,9122 to 0,9990
R squared	0.9811
P value	
P (two-tailed)	0.0001
P value summary	***
Significant? (alpha = 0.05)	Yes
Number of XY Pairs	6

Supplementary Figure 3. Results of optimization of the bacteria opsonization step by conventional AIA. **(A)** The graph illustrates the relationship between the pooled human serum dilution factor, on the X axis, and the absolute number of internal and adherent bacteria when infecting HT-29 cells with *S. sonnei* 53G OAg- strain, on the Y axis, with and without the 30 min opsonization step. **(B)** The table reports the results of the Pearson r test which points at a statistically significant correlation between the two conditions tested in the graph in A.

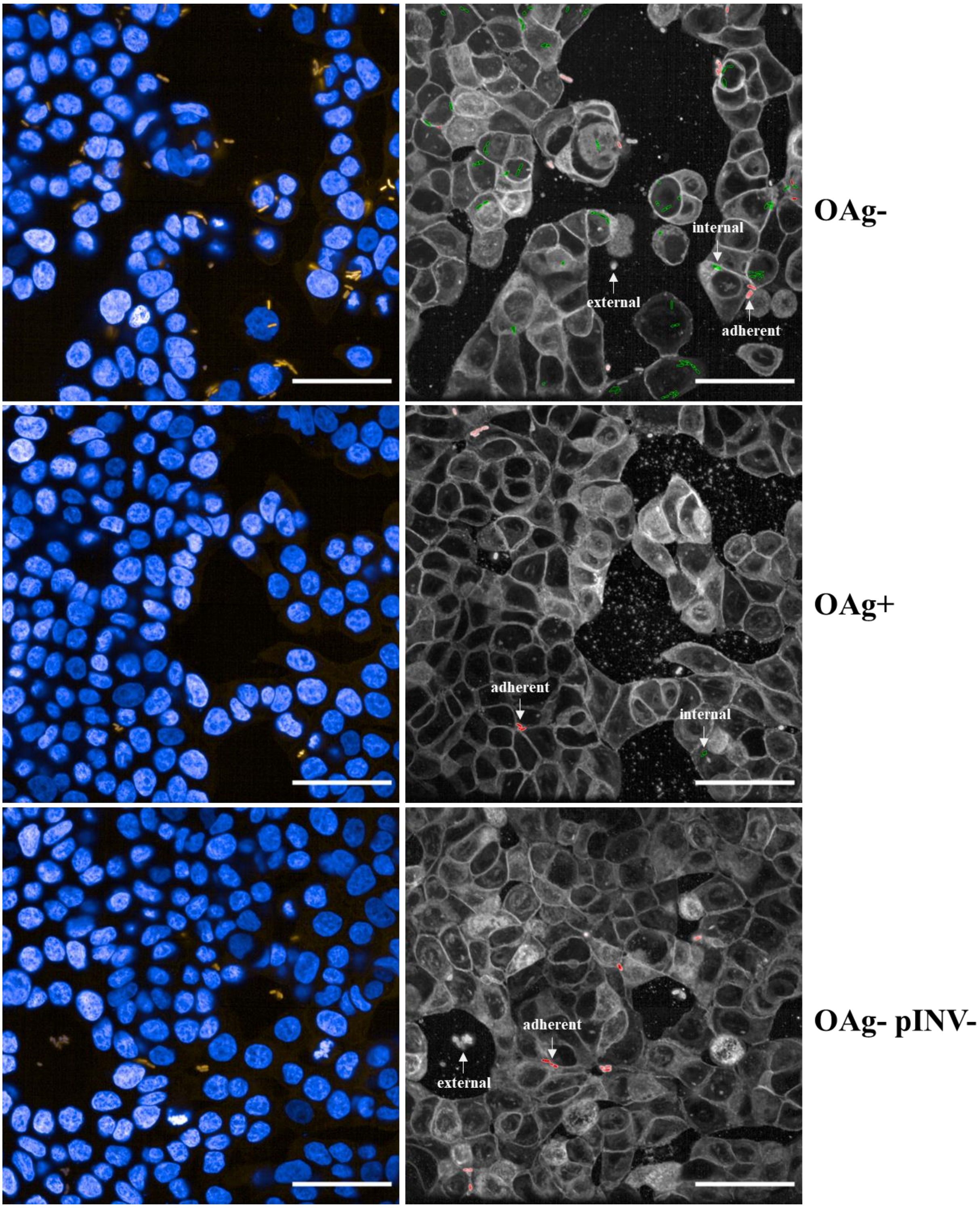

Supplementary Figure 4. Comparison between the infection rates of *S. sonnei* 53G OAg-, OAg+ and OAg- pINV- strains at MOI 100 as visualized at the microscope. The images represent the 3 different strains infecting HT-29 cells without adding any serum or mAb. Nuclei appear blue due to DAPI staining, sfmCherry-expressing bacteria are in yellow. In the corresponding segmented panels (on the right), external bacteria are those previously filtered out and thus not assigned to any color by the Harmony software, while the internal and the adherent bacteria are depicted in green and red, respectively (see Fig. 2 A, panel 8 “Internal and adherent bacteria”). Scale bars correspond to 50 µm.

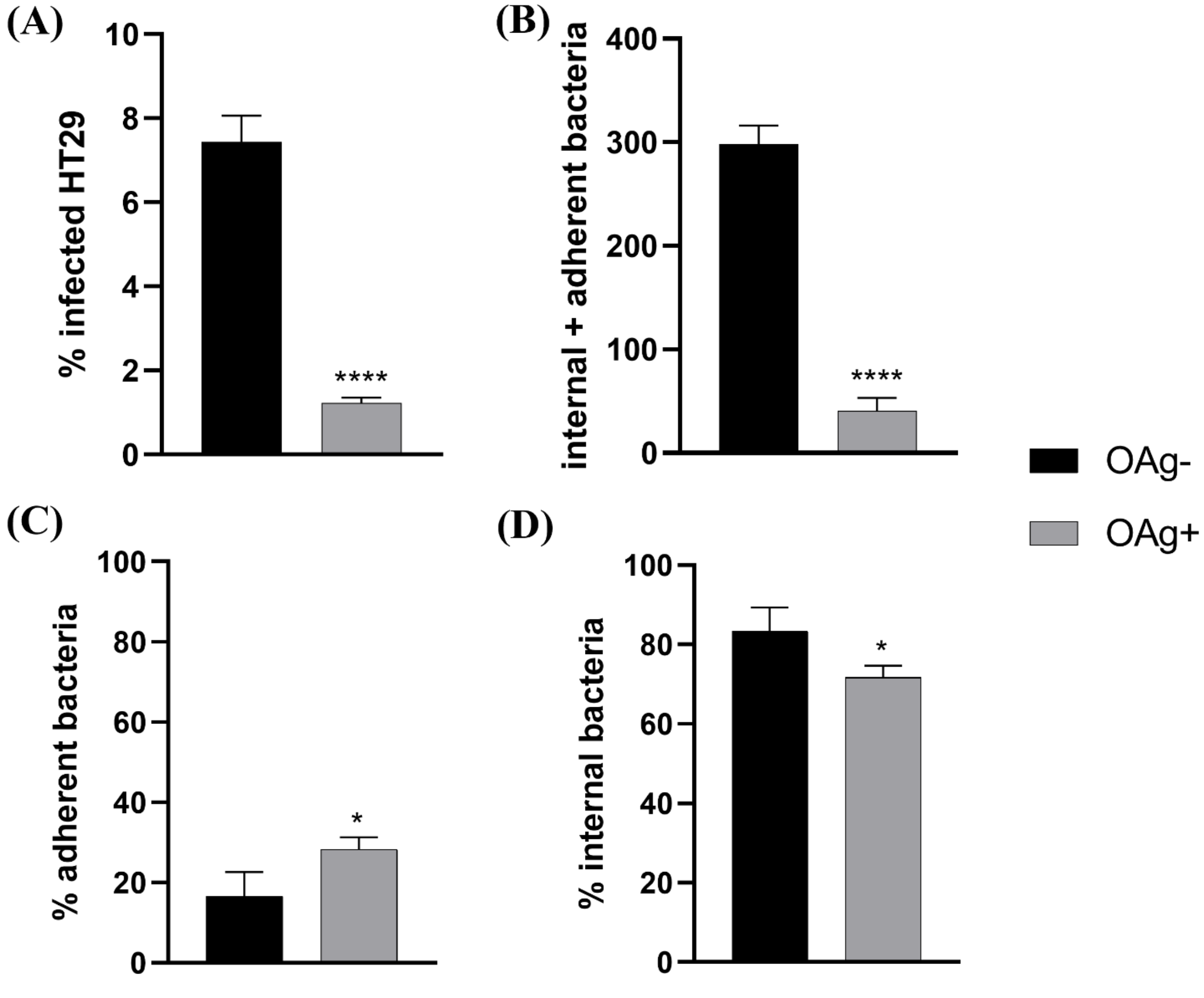

Supplementary Figure 5. Comparison among the infection rates of *S. sonnei* 53G OAg- and OAg+ strains at MOI 30. The 4 graphs show the differences between these 2 strains in terms of percentage of infected cells **(A)**, absolute number of internal and adherent bacteria **(B)**, and the percentages of adherent **(C)** and internal **(D)** bacteria. Unpaired t test was used to compare the OAg- and the OAg+ strains, with significant differences denoted by **** corresponding to a p-value (P) < 0.0001, and * to P ≤ 0.05.
